# Amphistome Species in Cattle in South Coast of Caspian Sea

**Published:** 2012

**Authors:** SZ Coskun, A Eslami, A Halajian, A Nikpey

**Affiliations:** 1 Dept. of Parasitology, Faculty of Veterinary Medicine, University of Uludag, Turkey; 2 Dept. of Parasitology, Faculty of Veterinary Medicine, University of Tehran, Tehran, Iran; 3 Dept. Parasitology, Veterinary Faculty, Science and Research Branch, Islamic Azad University, Tehran, Iran; 4 Dept. of Biology, Basic Sciences Faculty, University of Guilan, Rasht, Iran

**Keywords:** Parasitic Fauna, Amphistome, Cattle, Iran

## Abstract

**Background:**

Knowledge about the amphistomid fauna in Iranian domestic ruminants depends on the studies conducted almost 30 years ago. The last situation in cattle is introduced here in the provinces in south coast of Caspian Sea.

**Methods:**

Amphistomid species were collected from cattle at slaughter houses of the provinces Gilan and Mazanderan in 2010. Median sagittal sections were prepared by the conventional method. Species were identified by the histomorphological pecularities of the muscular argans.

**Results:**

Five amphistomid species, *Paramphistomum cevri*, *P. gotoi*, *Calicophoron calicophorum*, *Carmyerius spatiosus and Gastrothylax compressus*, recovered. *Calicophoron calicophorum* is a new species for Iran. Criteria used in identification of the species were illustrated.

**Conclusion:**

Iran has a rich amphistomid fauna and mostly under the influence of oriental conditions.

## Introduction

The last comprehensive study on the amphistome species of Iranian ruminants was published almost 30 years ago by Sey and Eslami (1). In that study, 10 amphistomid species were described in ruminants in detail and illustrated as *Paramphistomum cervi, P. gotoi, P. gracile, P. Microbothrium* (now *Calicophoron microbothrium), Calicophoron papillosum, Gastrothylax crumenifer, G. compressus, Gigantocotyle explanatum* and *Orthocoelium scoliocoelium*). Additionally, *Cotylophoron cotylophorum* and *Orthocoelium orthocoelium* appeared in the list given by Rafyi et al. (2).

The present situation of amphistomid fauna of Iranian cattle in the provinces in south of Caspian Sea was determined in this study.

## Materials and Methods

Amphistomid species were collected from cattle, at slaughter houses of the provinces Gilan and Mazandaran in south coast of Caspian sea in 2010. Median sagittal sections from the samples fixed in 70% alcohol were prepared by the conventional method. Histomorphological features, especially those of muscular organs such as pharynx, genital opening and acetabulum were examined under the light of the literatures (3–5). Most important criteria used in the identification of the species were illustrated.

## Results

In the samples available five species recovered, including four species described by earlier authors. *Calicophoron calicophorum* was described for the first time in Iran.

### Paramphistomum cervi *Zeder, 1790*

**Fig. 1:**
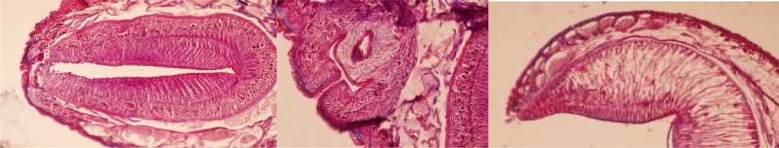
type of pharynx, *Gracile* type of genital opening and *Paramphistomum* type of acetabulum in *P. Cervi*

It has a *Liorchis* type of pharynx, *Gracile* type of genital opening and *Paramphistomum* type of acetabulum (Fig. 1). This species is closely related to *P. gotoi* which is also found in Iran. It differs from *P. gotoi* by having smaller papillae found in the pharynx and by the position of the blind ceaca which do not meet dorso medially.

### Paramphistomum gotoi *Fukui, 1922*

**Fig. 2:**
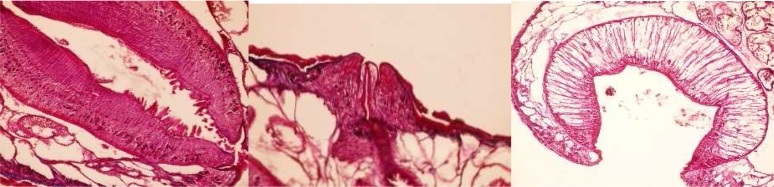
*Liorchis* type of pharynx, *Gracile* type of genital opening and *Paramphistomum* type of acetabulum in *P. gotoi*

It has a *Liorchis* type of pharynx, *Gracile* type of genital opening and *Paramphistomum* type of acetabulum (Fig. 2). This species is closely related to preceding one. It differs from *P. cervi* by having larger papillae found in the pharynx and by the position of the blind ceaca which usually meet dorso medially.

### Calicophoron calicophorum *( Fischoeder, 1901) Näsmark, 1937*

**Fig. 3:**
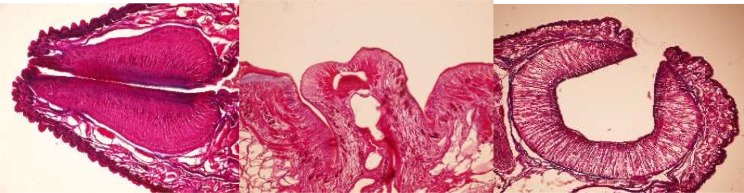
*Calicophoron* type of pharynx, *Calicophoron* type of genital opening and *Calicophoron* type of acetabulum in *C. Calicophorum*

It is a new species for Iran. It has *Calicophoron* type of pharynx, genital opening and acetabulum (note the absence of dorsal and ventral exterior circular 2 muscle units) (Fig. 3). This species is found characteristically in oriental region. Iran is the most western distributional area for *C. calicophorum* in Asia.

### Carmyerius spatiosus *Brandes, 1898*

**Fig. 4:**
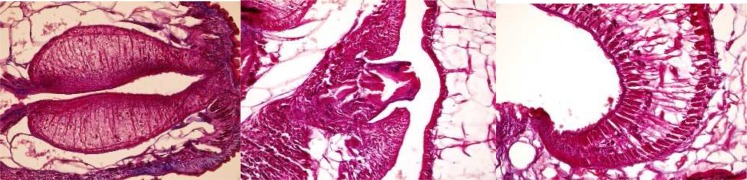
*Gastrothylax* type of pharynx, *Gracile* type of genital opening and *Gastrothylax* type of acetabulum in *C. spatiosus*

It has *Gastrothylax* type of pharynx, *Gracile* type of genital opening and *Gastrothylax* type of acetabulum (Fig. 4). Ventral pouch is triangular near to circular with blunt angles.

### Gastrothylax compressus Brandes, 1898

**Fig. 5:**
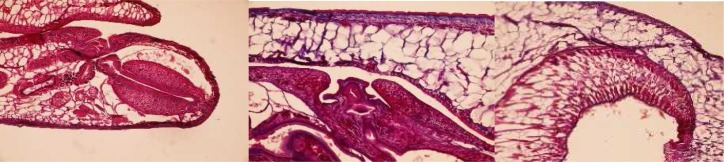
*Gastrothylax* type of pharynx, *Gracile* type of genital opening and *Carmyerius* type of acetabulum in *G. compressus*

It has *Gastrothylax* type of pharynx, *Gracile* type of genital opening and *Carmyerius* type of acetabulum (Fig. 5). Ventral pouch usually triangular with apex dorsally directed. This species accepted as synonym of *G. crumenifer* for a long time. However, they differs easily by having different type of acetabulum.

## Discussion

Findings of this study confirmed once more that Iran has a wide variety of amphistomid species as reported by previous researchers (1, 2, 4) and present authors. Five species have been identified in two provinces in the north of the country. Whereas, amphistomid fauna in ruminates in Turkey, the west neighbor country of Iran, consists only 3 species (*P. cervi, P. ichikawai*, *Calicophoron daubneyi*) as seen in Europe (6–8). Amphistomid fauna becomes richer in India, the east neighbor country (4, 9). *Paramphistomum cervi* and *P. gotoi* is wide spread in palaearctic region. Iran is the most west country where *C. calicophorum* and *G. compressus* recovered. *Carmyerius spatiosus* belongs to the Ethiopian region. South coast of the Caspian Sea is the most north point for this species (1). Its obviously seems that the amphistome fauna of Iran is strongly influenced by oriental and to a lesser extent by pale arctic and Ethiopian elements.

Distributional area of amphistomids is closely related to the climatic and microenvironmental conditions which affect the viability of the intermediate snail host. Further studies are necessary to elucidate the intermediate snail hosts of that species in the studied regions.
